# “Infection prevention and control idea challenge” contest: a fresh view on medical education and problem solving

**DOI:** 10.1186/s13756-020-0688-y

**Published:** 2020-02-07

**Authors:** Arash Arianpoor, Ahmadreza Zarifian, Emran Askari, Arash Akhavan-Rezayat, Mojtaba Dayyani, Amin Rahimian, Elahe Amini, Roya Amel, Aghigh Ziaeemehr, Walter Zingg, Mohammad Hasan Aelami, Didier Pittet

**Affiliations:** 1 Surgical Oncology Research Center, Mashhad University of Medical Sciences, Mashhad, Iran; 2 Mashhad Medical Microbiology Student Research Group, Mashhad University of Medical Sciences, Mashhad, Iran; 3 Orthopaedic Research Center, Mashhad University of Medical Sciences, Mashhad, Iran; 4 Student Research Committee, Mashhad University of Medical Sciences, Mashhad, Iran; 5 Nuclear Medicine Resident, Mashhad University of Medical Sciences, Mashhad, Iran; 6 Department of Neurology, McGovern Medical School, University of Texas Health Science Center at Houston, Houston, TX USA; 7 Infection Control Programme and World Health Organization Collaborating Centre on Patient Safety, University of Geneva Hospitals and Faculty of Medicine, 4 Rue Gabrielle-Perret-Gentil, 1211 Geneva, Switzerland; 8 Infection Control and Hand Hygiene Research Center, Imam Reza Hospital, Mashhad University of Medical Sciences, Shariati Square, Mashhad, Iran

**Keywords:** Infection control, Healthcare-associated infections, Medical education, Problem solving, Antimicrobial resistance, Surgical site infection, Hand hygiene, Healthcare economy

## Abstract

**Background:**

Healthcare-associated infections (HAIs) challenge modern medicine. Considering their high prevalence in Iran, we aimed to provide knowledge on the subject, and to teach about the importance of infection prevention and control (IPC) to a broad audience of pre-graduate healthcare professionals, focusing on education as the cornerstone of IPC.

**Main body:**

We invited Iranian medical students to present ideas on “how to reduce HAIs.” Projects were eligible if being original and addressing the call. Accepted projects were quality assessed using a scoring system. Forty-nine projects were submitted, of which 37 met the inclusion criteria. They had a mean score of 69.4 ± 18.3 out of the maximum possible score of 115. Four reviewers assessed the 37 projects for clinical applicability, impact on patient safety, and innovation, and selected the best 12 to compete at the 2nd International Congress on Prevention Strategies for Healthcare-associated Infections, Mashhad, Iran, 2018. The competition took place in three rounds. The selected teams presented their projects in the first round and debated one by one in a knockout manner, while the jury reviewed their scientific content and presentation skills. In the second round, the top 5 projects competed for reaching the final stage, in which the teams presented their ideas in front of a panel of international IPC experts to determine the first three ranks. At the end of the contest, the participants gained valuable criticisms on how to improve their ideas. Moreover, by its motivating atmosphere, the contest created an excellent opportunity to promote IPC in medical schools.

**Conclusions:**

Using innovation contests in pre-graduates is an innovative education strategy. It sensitizes medical students to the challenges of IPC and antimicrobial resistance and drives them to think about solutions. By presenting and defending their innovations, they deepen their understanding on the topic and generate knowledge transfer in both ways, from students to teachers and vice versa.

## Background

Healthcare-associated infections (HAIs) and antimicrobial resistance are among the most serious challenges in modern medicine, affecting both patients and healthcare workers (HCW). The global HAI prevalence has been reported to be up to 15.5% in low and middle income countries, and the number is growing [1–5]. On the other hand, 35 to 55% of HAIs can be reduced by applying multimodal infection control measures [6].

Many barriers affect best practice of infection prevention and control (IPC) measures, but the lack of knowledge on implementing IPC strategies and low compliance with best practice guidelines are among the most important. Various ideas to increase compliance toward IPC guidelines have been published, most of which include educational programmes taking into account local contexts [7]. A Chinese group reported their positive experience in implementing an educational intervention among nurses that significantly improved knowledge, practice, and behaviour related to universal precaution measures [8]. A one-day course on IPC practices regarding central venous catheter (CVC) insertion for medical students and physicians in the US resulted in a significant decrease of CVC-associated infections 18 months after the event [9]. Education both for under- and postgraduates is key, and the World Health Organization (WHO) emphasized the importance of formal education in IPC in their guidelines [10].

Ex-cathedra education or training focusing on knowledge alone is not sufficient for behaviour change, and the implementation of IPC strategies is most effective when applying a multimodal strategy [10, 11]. The multimodal approach of the Geneva hand hygiene model using system change (move from hand washing to hand rubbing), educational tools, reminders in the work environment, active participation of staff, surveillance and feedback, as well as credible involvement of leaders not only improved hand hygiene compliance but also reduced HAI and cross-transmission of multi-drug-resistant organisms [12]. Consistently, WHO developed and promoted a multimodal implementation strategy for hand hygiene improvement that are feasible, sustainable, and adaptable to different contexts. The strategy was reported to improve both knowledge of and compliance with hand hygiene in both different geographical regions and different healthcare settings [13, 14]. Multimodal strategies were identified as one of the key components for effective IPC strategies in acute care [15], and also were identified as one of the WHO core components for IPC, both at institutional and national levels [10–14, 16, 17].

Compliance with best practice procedures differs among healthcare professions, with doctors often be less compliant toward basic IPC measures such as hand-hygiene [18]. Thus, more effort should be invested in improving compliance of doctors with best practice procedures. An integrated IPC curriculum during medical school would help to achieve this goal. Although multimodal implementation strategies are the most effective way to improve IPC practices [13, 15, 17], it is difficult to communicate this concept to medical students.

To overcome the challenge of sensitizing medical students to the burden of HAI in Iran [3, 4, 19], and delivering concepts of best practice in a complex multidisciplinary work context, to which students have not been fully exposed, the Mashhad Medical Microbiology Student Research Group [MMMSRG] organized workshops, campaigns, and conferences on IPC [2]. Here, we describe the “*Infection Prevention and Control Idea Challenge”* contest, which was organized as part of the “2^nd^ International Congress on Prevention Strategies for Healthcare-Associated Infections” in April 2018 in Mashhad, Iran.

### The “Infection Prevention and Control Idea Challenge” contest

The “Infection Prevention and Control Idea Challenge” was advertised and promoted by MMMSRG on January 2, 2018, on the congress website and via social media. Medical students, who were known to MMMSRG, were directly addressed to act as ambassadors for the contest at their local medical school. This promotional activity by itself initiated a movement to organize IPC campaigns and workshops in different medical schools, to teach medical students the basics of IPC, and to motivate and prepare them to develop and submit projects to the contest.

**Fig. 1:**
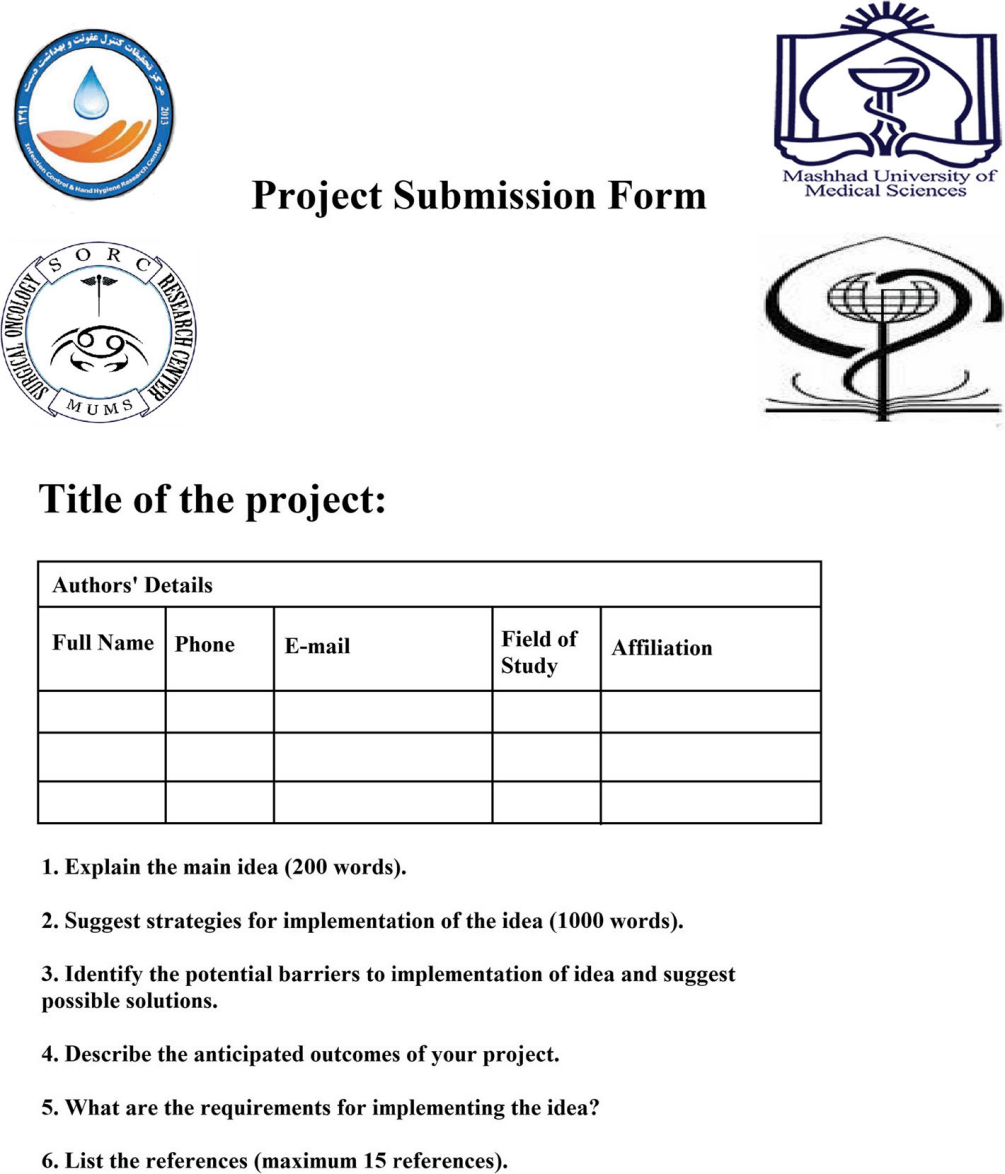
Project submission form of the “Infection Prevention and Control Idea Challenge” contest

A panel of IPC experts, journal editors, and reviewers designed formats for collecting ideas (Fig. 1), assessing their eligibility for oral presentation (Table 1) and evaluating the projects and their presentations by the participating students (Table 2). Projects had to be submitted using a pre-defined format (Fig. 1), addressing one of the four main areas of the contest:

1. Adherence to hand hygiene guidelines: “How can we remove the barriers (to appropriate adherence to hand hygiene guidelines)?”
2. Antibiotic stewardship: “How do we manage antibiotic misuse and overuse?”
3. Post-surgical and procedure-related infections: “What is the best solution (to prevent post-surgical and procedure-related infections)?”
4. The role of society and economy in IPC: “How can we advertise and get a return on investment in IPC?”

**Table 1:**
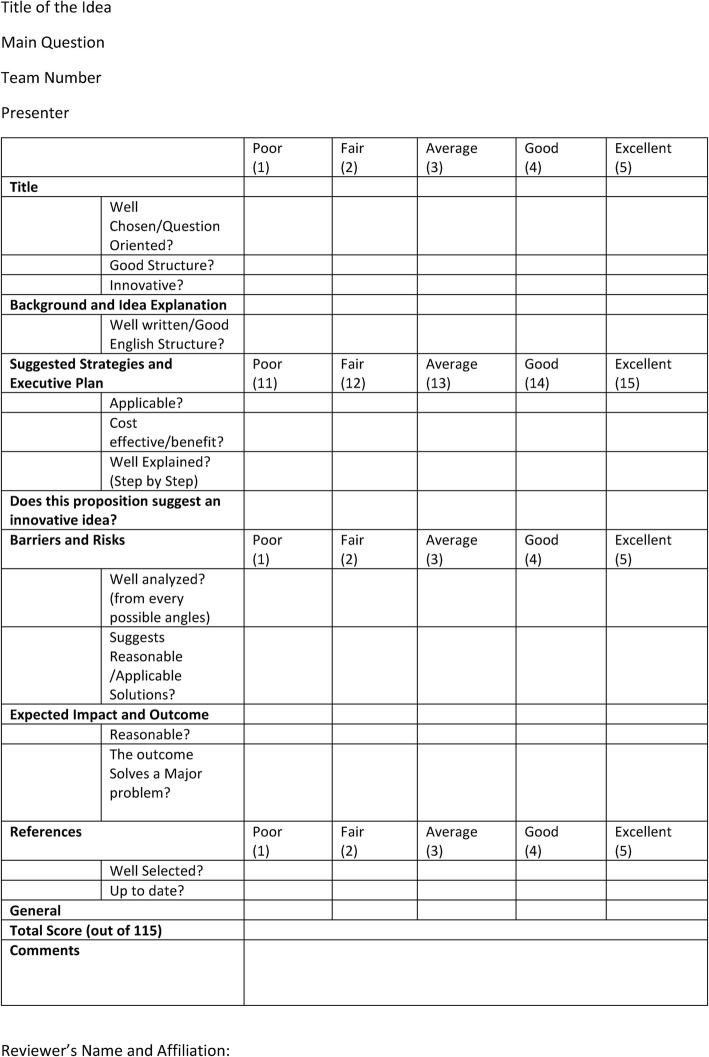
The primary evaluation rubric of the “Infection Prevention and Control Idea Challenge” contest

**Table 2:**
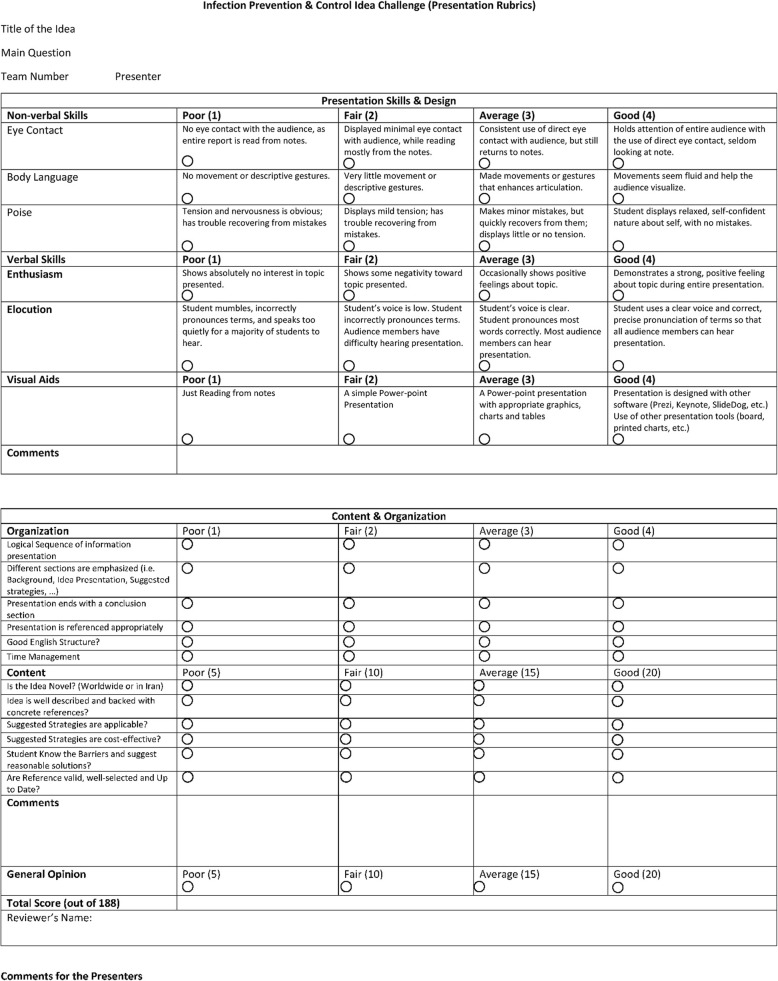
The final evaluation rubric of the “Infection Prevention and Control Idea Challenge” contest

With 49 submitted projects, the resonance of the call was unexpectedly high. A single reviewer reviewed the projects to check formal eligibility. Twelve projects were excluded for plagiarism or not being submitted in the correct format. The 37 remaining projects were evaluated and scored by four reviewers (2 national and 2 international IPC experts) based on pre-determined evaluation criteria (Table 1).

The mean scores of the projects are summarised in Table 3. Of a maximum of 115, the mean (± standard deviation) score of the 37 accepted projects was 69.4 (± 18.3), ranging from 21.0 to 92.3 (Table 3). Based on consensus among the organisers of the contest, the 12 projects with the highest scores were selected to be presented at the conference. Details of these projects are summarized in Table 4.

**Table 3:** Titles and attributed scores of the received ideas

No	Title of the Project	Mean score of the primary review (out of 115)	Mean score of the 1st round (out of 188)	Mean score of the 2nd round (out of 188)	Mean score ± SD of the final presentation
1	The next generation of antiseptics (revised: The next generation of antiseptic *Containers*)	89.5 ± 10.60	151.2 ± 2.28	155 ± 5.78	160.5 ± 19.84
2	An automatic hand hygiene monitoring system	87 ± 24.04	161.2 ± 6.41	163.2 ± 6.37	153 ± 12.02
3	Using the pocket chart to reduce antibiotic resistance	88 ± 15.55	146.6 ± 5.98	153 ± 6.59	142. 5 ± 22.48
4	Post-operation patient care to prevent and control infections through the use of a mobile application	92.25 ± 7.42	141.8 ± 5.49	135.6 ± 4.61	N/A
5	Mobile handrub dispenser	88 ± 18.38	137.2 ± 5.71	140.2 ± 5.06	N/A
6	Developing an integrated antibiotic monitoring and management governmental system to reduce resistance to antibiotics	90.5 ± 14.84	130.6 ± 4.56	N/A	N/A
7	Prevention of implant-associated infections by using electrospun nanofibers	85 ± 2.82	122 ± 6.63	N/A	N/A
8	Produce and use of new yarn stitches based on silver nanoparticles	84.5 ± 17.67	122.2 ± 4.49	N/A	N/A
9	Nanotechnology: the open way of infection control, prospects	84.5 ± 13.43	115.2 ± 5.06	N/A	N/A
10	Bacteriotherapy for wound healing	82.5 ± 10.60	117.6 ± 3.04	N/A	N/A
11	How can we manage healthcare-associated infections in hospitals?	82.5 ± 20.50	105.8 ± 6.41	N/A	N/A
12	Combination strategy (restriction-education) for antibiotic stewardship program	79.5 ± 9.01	105.4 ± 7.02	N/A	N/A
13	Appropriate education of hand hygiene for children in schools	79 ± 21.21	N/A	N/A	N/A
14	Prevention and control of vaginal infections associated with swimming pools	78 ± 5.65	N/A	N/A	N/A
15	Establishing permanent workshops on skills and ideas about prevention and control of infection in high schools	77.5 ± 0.70	N/A	N/A	N/A
16	Ways to decrease the surgical site infection rate in Iran	75.5 ± 9.19	N/A	N/A	N/A
17	Side effects after surgery and recovery (make some proprietary suture)	75.5 ± 20.50	N/A	N/A	N/A
18	Training ways to control and prevent oral infections in elementary students	75 ± 12.72	N/A	N/A	N/A
19	Antimicrobial resistance, as big a risk as terrorism	75 ± 12.72	N/A	N/A	N/A
20	Strategies for improving hand hygiene, as a preventive measure against hospital-acquired infections	75 ± 16.97	N/A	N/A	N/A
21	Students as WHO health representatives in Iranian schools; to modify previous strategies for better ones	73.5 ± 3.53	N/A	N/A	N/A
22	Early diagnosis of prosthetic joint infections: focus on biomarkers	70.5 ± 10.60	N/A	N/A	N/A
23	Prevention of infection by diagnosing cancer through biomarker levels and dielectric qualities as a minimally invasive and novel method	68.5 ± 7.77	N/A	N/A	N/A
24	Placebo prescription and home remedies; a novel but ignored method in Iran	68 ± 7.07	N/A	N/A	N/A
25	Online medical network and acute infections control	67.5 ± 7.77	N/A	N/A	N/A
26	A traditional solution for antibiotic overuse	67.25 ± 18.03	N/A	N/A	N/A
27	Monitoring policy for antibiotics therapy	65 ± 0	N/A	N/A	N/A
28	Adherence to hand hygiene guidelines	60.5 ± 2.12	N/A	N/A	N/A
29	Fixing policies and education	56.5 ± 31.81	N/A	N/A	N/A
30	Our hands, where all things begin	55.5 ± 7.77	N/A	N/A	N/A
31	Antibiotic stewardship: How do we manage antibiotic misuse and overuse?	55.25 ± 15.20	N/A	N/A	N/A
32	Surgery without infection	48.5 ± 13.61	N/A	N/A	N/A
33	Controlling respiratory infections in garrison	46 ± 14.31	N/A	N/A	N/A
34	Increasing the level of applied scientific information of patients	40 ± 34.11	N/A	N/A	N/A
35	Penicillin vs natural herbs esp. turnip	35.25 ± 33.58	N/A	N/A	N/A
36	Comparison of the effects and side effects of amoxicillin and penicillin on teeth	24.25 ± 32.88	N/A	N/A	N/A
37	Medical universities as an economic corporation	21 ± 23.51	N/A	N/A	N/A

**Table 4:** Details of the 12 selected ideas presented orally

Rank	Title	Main Idea
1	The next generation of antiseptics (revised: The next generation of antiseptic *Containers*)	The main innovative idea of this team was to design an alcohol-based handrub container connected to a counter. This counter would not only show the number of times it is used by the HCWs, but it would also show an inspirational scientific fact about the importance of handrubs in hand-hygiene, hospital-acquired infections, and IPC practices. The main concept behind this idea was that increasing hand-hygiene adherence needs motivation and education.
2	An automatic hand hygiene monitoring system	This project was based on “monitoring and surveillance” as an integral part of IPC. The innovative aspect of this idea was designing an electronic badge for HCWs that is connected to the handrub dispenser and they suggested an integrated plan consisted of three parts:
		1. All entrance doors should be connected to a handrub dispenser that will work as a key to the door, so the doors will open only if the handrub dispenser is used by the HCWs. Besides, the use of handrubs will be recorded by the electronic badge as a monitoring device.
		2. An infection control area will be defined around patients’ beds and if HCWs enter this area without using the handrub dispenser, an alarming signal (beeping sound, alarming light, or a vibration) via their badge will notify them to wash their hands before approaching to the patient.
		3. The data collected by the badges will be used to monitor the hand-hygiene adherence among HCWs and may be used as a reward-punishment system to enforce this behaviour.
3	Using the pocket chart to reduce antibiotic resistance	This project was focused on antibiotic stewardship. The main idea was to design a user-friendly chart, called “Pocket Chart”, including indications for starting antibiotics and antibiotic duration as well as a detailed report on antibiotic resistance patterns. Although the main idea of using guidelines for antibiotic prescription is not novel, local guidelines for different conditions are lacking in Iran. The team suggested that their Pocket Chart would be customized not only for each different hospital ward but also for different areas of the city for outpatients.
4	Post-operation patient care to prevent and control infections through the use of a mobile application	The main idea of this project was to design an app to monitor patients after surgery and develop a platform for easy communication between patients and doctors. The app would have 4 parts, i.e. patient’s profile, notification panel, messaging, and question and answer part. The app would be supported by an online server containing medical records of the patients. The application would also make possible for different patients to communicate with each other and share their experiences. The application would gather information on patients’ condition after surgery and notify their doctors in case any red flag signs occur.
5	Mobile handrub dispenser	This project focused on increasing the accessibility of the alcohol-based handrub solutions by designing a wearable handrub dispenser. The design included a wearable dispenser designed as a bracelet or armlet with a refillable container and a handrub container mounted on the walls that could be used to refill the bracelet/armlet.
6	Developing an integrated antibiotic monitoring and management governmental system to reduce resistance to antibiotics	This idea suggested an integrated monitoring and management system that oversees the antibiotic prescription and usage. This system includes three parts:
		1. Developing a two-part visit strategy for doctors, in which all patients with an indication for antibiotic use will be prescribed with the appropriate antibiotic. After a few days, the patient’s condition and response to antibiotic therapy will be evaluated
		2. Developing an electronic system to record the amount of antibiotics prescribed for each patient and monitor their antibiotic use
		3. Making strict rules for selling over-the-counter antibiotics Although these strategies are common in many developed healthcare system, in Iran we do not have an integrated system for antibiotic prescription monitoring
7	Prevention of implant-associated infections by using electrospun nanofibers	In this project, the contestants aimed to develop a polymeric dressing with tunable drug release to be used in orthopaedic surgeries. The designed dressing would be made through electrospinning process and contain desired antibiotics. They suggested that their dressing would be applied on the implant site before closing the wound in the operation room.
8	Production and use of new yarn stitches based on silver nanoparticles	This team designed suture threads coated with silver nanoparticles to prevent surgical site infection.
9	Nanotechnology: the open way of infection control, prospects	This team designed a urinary catheter coated with silver nanoparticles to prevent urinary tract infections.
10	Bacteriotherapy for wound healing	This project was mainly focused on a wound dressing that contains *Lactobacillus rhamnosus* GG and *Lactobacillus plantarum* isolates and prevents infections related to diabetic foot.
11	How can we manage healthcare-associated infections in hospitals?	Designing an electronic device containing patients’ files and charts to replace conventional charts was the focus. The device would scan HCWs’ hands and give access only to those with low bacterial contamination.
12	Combination strategy (restriction-education) for antibiotic stewardship program	This team developed an executive plan for antibiotic stewardship in three steps: A. developing an electronic medical record software for monitoring antibiotic use, B. imposing strict rules with severe fines for over-the-counter antibiotic selling and C. mass education programs for general population.

Three rounds of project presentations were organised during the conference to select three winners. The first round grouped the selected 12 teams (Table 4) into six pairs based on the proximity of their project ideas. Each team competed to the allocated other team. They were allowed to see the abstracts of their competitors before presentation for better criticism. Each project was discussed for 15 min: the team presented the main concepts of their project in the first 5 min; the competitor team had the opportunity to express criticism in the second 5 min; the presenting team had time to address questions and concerns raised by the competitors in the last 5 min. Content and presentation were assessed by a jury of 5 general practitioners using a pre-defined evaluation form (Table 2). The general practitioners have worked and published in the field of IPC and infectious diseases, and they have worked in both public and private hospitals. Of a maximum of 188, the mean (± standard deviation) score was 129.7 (± 18.0), ranging from 105.4 to 161.2 (Table 3). Although it was planned to select one team from each pair for the second round, only the top five projects were selected because some presentations were poor and the knock-out strategy would have eliminated good projects. The teams of the five selected projects had to modify their project based on the comments raised by the jury members in preparation for the second round.

In the second round, the teams had 5 min to present their revised projects, which was followed by two minutes during which all other competitors could express criticism and concerns. The presenting team then had another 5 min to address criticism and raised concerns. The same jury from the first round also assessed the projects in the second round, using the same scoring form (Table 2). The top three projects were invited to be presented in the final round on the last day of the conference to a mixed national (1) and international (3) expert panel (Table 3). The experts ranked the projects using the pre-defined evaluation form (Table 2).

Table 4 summarises the 12 projects presented at the conference in descending order of the scores. The three winning projects are summarised below:

1. The next generation of alcohol-based handrub containers: The main innovative idea was to design an alcohol-based handrub container connected to a counter. This counter would not only show the number of times the HCWs use it, but it would also display an inspirational scientific fact about the importance of hand-hygiene, HAIs, and IPC practices. The main concept behind this idea was that behaviour change toward hand hygiene needs not only education but also motivation.
2. An automatic hand-hygiene monitoring system: This project was based on “monitoring and surveillance” as an integral part of IPC. The innovative aspect of this idea was an electronic badge connected to the handrub dispenser, which grants entrance to patient rooms or triggers an alarm:
  - The dispenser connected to the patient room works as a door key, so the door will open only if the HCW uses the handrub dispenser.
  - An infection control area is defined around the patient (patient zone); if a HCW enters this zone without using the handrub dispenser, an alarm (beeping sound, flashing light, or vibration) issued by the personal badge notifies the HCW to perform hand hygiene before approaching the patient.
  - The data collected by the badge will be used to monitor individual hand-hygiene compliance and may feed a reward-punishment system.
3. Using a pocket chart to reduce antibiotic resistance: This project focused on antibiotic stewardship. The main idea was to design a user-friendly chart called “Pocket Chart,” which displays indications for starting and stopping antibiotics combined with a detailed report on local antibiotic resistance patterns. Although the main idea of using guidelines for an antibiotic prescription is not novel, guidelines taking into account local antimicrobial resistance are lacking in Iran. The team suggested that their “Pocket Chart” would be customized not only for each hospital but also for outpatient care.

### Outcome and impacts

Although HAIs are a significant challenge for healthcare systems, it is believed that more than 30% of HAIs could be prevented by correct IPC measures [6, 20]. Unfortunately, adherence to best practices and guidelines is very low [10, 21]. Even in an environment with numerous promotional campaigns on hand hygiene, compliance depends more on “peer pressure and the perception of high self-efficacy” than on reasoning [22]. Given that medical doctors repeatedly have been reported with low compliance to IPC measures such as hand-hygiene, it seems essential to integrate IPC in the curriculum of medical schools. However, as communicating multimodal behaviour change strategies to medical students might be challenging, an IPC contest can motivate students to engage in IPC and to invent innovative solutions. This adds to the main goal of medical education to have knowledgeable, skilled and up-to-date healthcare professionals who choose patients’ interests over their own [23]. However, patient safety, and particularly IPC is a long-term investment and maintaining professional expertise needs continuous learning also after graduation [24].

Overall, our IPC idea challenge contest had four main outcomes:

1. Sensitizing medical students for the problems around IPC and antimicrobial resistance
2. Integrating IPC and antimicrobial resistance in the medical curricula
3. Fostering the creation of workshops and educational sessions on IPC and antimicrobial resistance
4. Encouraging medical students to be solution- rather than problem-oriented when thinking about challenges in IPC and antimicrobial resistance

By inviting the students to participate in this programme, we challenged them to get interested in IPC but also to think critically in terms of basic concepts, strategies, interventions, and implementation strategies. The high number of submitted projects of good quality combined with high motivation and investment by the medical students was a success, and thus, contests may be an effective educational strategy.

We reviewed all projects and provided feedback to all participants to help them to improve their ideas and make them more practical and implementable. The contest promoted education in both ways: from students to peers and experts, and from experts to students. The contest also provided an interactive environment via debates, in which participants learned to review projects and articulate concerns and constructive criticism.

Another important outcome of our contest was linking students to their supervisors for identifying innovative ideas in IPC and to prepare a competitive project. The contest helped medical students to do both learn about a problem and come up with solutions. The opportunity to compete with peers from other universities in Iran, and attending an international conference probably were important incentives and drivers for investing in the contest.

## Conclusions

Using innovation contests in pre-graduates is an innovative education strategy. It sensitizes medical students to the challenges of IPC and antimicrobial resistance and drives them to think about solutions. By presenting and defending their innovations, they deepen their understanding on the topic and generate knowledge transfer in both ways, from students to teachers and vice versa.

## Data Availability

The datasets used and/or analysed during the current study are available from the corresponding author on reasonable request.

## Abbreviations

AMR: Antimicrobial resistance
CVC: Central venous catheter
HAI: Healthcare-associated infections
HCW: Healthcare workers
IPC: Infection prevention and control
MMMSRG: Mashhad Medical Microbiology Student Research Group
WHO: The World Health Organization
